# Mindfulness-Based Mobile Health to Address Unhealthy Eating Among Middle-Aged Sexual Minority Women With Early Life Adversity: Mixed Methods Feasibility Trial

**DOI:** 10.2196/46310

**Published:** 2023-09-26

**Authors:** Shufang Sun, William Nardi, Matthew Murphy, Ty Scott, Frances Saadeh, Alexandra Roy, Judson Brewer

**Affiliations:** 1 Department of Behavioral and Social Sciences Brown University Providence, RI United States; 2 Mindfulness Center at Brown Brown University Providence, RI United States; 3 School of Public Health Brown University Providence, RI United States

**Keywords:** mobile health, mindfulness, obesity, sexual minority women, early life adversity, cell phone, mobile phone

## Abstract

**Background:**

Sexual minority women (lesbian, gay, bisexual, pansexual, queer, and other nonheterosexual women) remain considerably underrepresented in health research despite being at a higher risk for diabetes and obesity as well as stigma and psychological distress than their heterosexual peers. In addition, early life adversity (ELA) is prevalent among sexual minority women, which further increases risks for obesity, psychological distress, and poor cardiovascular health. App-based mindfulness interventions are potentially promising for this group in mitigating the adverse health effects of ELA, reducing food craving and unhealthy eating, addressing the risks associated with obesity.

**Objective:**

This mixed methods feasibility trial aimed to test a mindfulness-based mobile health approach for middle-aged sexual minority women (aged 30-55 years) with ELA and overweight or obesity (BMI ≥25 kg/m^2^) to improve health outcomes.

**Methods:**

The single-arm trial was advertised on social media and various lesbian, gay, bisexual, transgender, and queer web-based groups. At baseline, after the intervention (2 months), and at the 4-month follow-up, participants completed assessments of primary outcomes (food craving, emotional eating, and weight via a mailed scale) and secondary outcomes (depression, anxiety, mindfulness, and emotion dysregulation). A standardized weight measure was mailed to participants for weight reporting. Feasibility and acceptability were assessed after the intervention via surveys and semistructured exit interviews.

**Results:**

We screened 442 individuals, among which 30 eligible sexual minority women (mean age 40.20, SD 7.15 years) from various US regions were enrolled in the study. At baseline, 86% (26/30) and 80% (24/30) of participants had elevated depressive and anxiety symptoms, respectively. Among the 30 enrolled participants, 20 (66%) completed all intervention modules, 25 (83%) were retained at the 2-month follow-up, and 20 (66%) were retained at the 4-month follow-up. None reported adverse effects. From baseline to the 4-month follow-up, large effects were found in food craving (Cohen *d*=1.64) and reward-based eating (Cohen *d*=1.56), whereas small effects were found with weight (Cohen *d*=0.20; 4.21 kg on average). Significant improvements were also found in the secondary outcomes (depression, Cohen *d*=0.98; anxiety, Cohen *d*=0.50; mindfulness, Cohen *d*=0.49; and emotion dysregulation, Cohen *d*=0.44; all *P*<.05). Participants with higher levels of parental verbal and emotional abuse were particularly responsive to the intervention. Participants reported that the program aligned with their goals and expectations, was easy to use, and facilitated changes in eating behavior and mental health. Barriers to engagement included the need for diverse teachers, individualized support, and body positive language.

**Conclusions:**

This early phase feasibility trial provides proof-of-concept support for a mindfulness mobile health approach to improve obesity-related outcomes among sexual minority women and warrants a larger randomized controlled trial in the future. The findings also suggest the need to address trauma and psychological health when addressing weight-related outcomes among sexual minority women.

## Introduction

### Obesity, Cardiovascular Health, and Sexual Minority Women

Obesity is a rising public health issue in the United States, affecting 42% of adults [1]. The prevalence of obesity is highest among middle-aged adults than young and older adults [1]. Obesity is associated with a greater risk for cardiovascular disease, diabetes, cancer, and mortality [2,3], leading to a significant health care cost of US $173 billion annually in the United States [4]. Compared with heterosexual women, sexual minority women (lesbian, bisexual, and queer women) experience a significantly higher risk of being overweight (BMI 25-29.9 kg/m^2^) and obese (BMI ≥30 kg/m^2^), as well as linked conditions such as prediabetes and diabetes [5-7]. For instance, an analysis of the 2014 to 2015 Behavioral Risk Factor Surveillance System (n=136,878) data found that bisexual and lesbian women had 31% and 17% higher risks of obesity than their heterosexual peers, respectively [6]. Sexual minority women are also disproportionally affected by psychological distress (depression and anxiety), which can further contribute to craving and emotional eating [6,8]. Given these disparities, the American Heart Association recently issued a statement calling for interventions to address cardiovascular health in lesbian, gay, bisexual, transgender, and queer (LGBTQ+) adults, including obesity-related health outcomes among sexual minority women [9].

Despite this call to action, sexual minority women are underrepresented in research. A recent analysis of the funding portfolio of sexual and gender minority research projects supported by the National Institute of Health in 2020 found that 69.6% of the projects focused on sexual minority men, with only 8.3% focusing on lesbian women and 17.2% focusing on bisexual people (sex or gender unspecified) [10]. In addition to the overall research deficits, interventions focusing on reducing obesity risk among sexual minority women are lagging. To our knowledge, only 2 intervention studies have been published that addressed healthy eating among older sexual minority women with overweight or obesity [11,12]. More intervention research targeting unhealthy eating and psychological distress among sexual minority women is needed to improve population health and promote health equity.

### Early Life Adversity as a Risk Factor

Meanwhile, individuals who have had early life adversity (ELA)—particularly psychosocial forms of ELA such as physical and emotional abuse from caregivers and peers, sexual violence, and neglect (physical or emotional)—are also at risk for obesity and worse cardiovascular health during adulthood [13,14]. In particular, middle age is a pivotal stage in which ELA starts to assert its role on an individual’s health trajectory. Detrimental health effects associated with ELA exposure are more prevalent and disabling in middle age [15,16], yet there is a lack of intervention research in this area. Furthermore, no intervention exists that aims to address unhealthy eating among adults with a history of ELA. In response to the scarcity of intervention research, the National Institute on Aging funded the Reversibility Network to foster the translation of scientific knowledge to interventions aimed at remediating the adverse health effects of ELA in mid-to-late adulthood. This pilot study is informed and supported by the Reversibility Network.

### The Potential of Mindfulness Mobile Health and This Study

Mindfulness-based interventions may offer promise for middle-aged sexual minority women with overweight or obesity for mitigating the adverse health effects of ELA as well as reducing food cravings and unhealthy eating. A review of mindfulness-based interventions for obesity-related unhealthy eating also supports the use of mindfulness in changing eating behaviors, particularly emotional eating [17]. Mindfulness may help to reduce craving, as suggested by a review of 30 experimental studies that found that mindfulness can be effective in both craving reduction and the amount of food consumed due to craving [18]. Coupled with mobile health (mHealth) delivery, this provides a potential solution to reach and engage sexual minority women, an underserved and hidden population. As a relatively low-cost approach, mindfulness-based mHealth could be a useful strategy to help with the adverse health consequences of ELA exposure [19]. However, the feasibility and safety of a mindfulness approach to reduce cravings and emotional eating are yet to be understood.

This study examined the feasibility of a mindfulness-based mHealth app–based program (*Eat Right Now*) to address cravings, reward-based eating, and weight loss among middle-aged sexual minority women with overweight or obesity and have a history of ELA. The primary aim was to understand the feasibility, acceptability, and safety of a remotely conducted mindfulness app intervention in this population. Secondary aims included exploring the preliminary effects of the intervention from baseline to follow-up, as well as the baseline ELA characteristics associated with the intervention effect.

## Methods

### Participants and Enrollment Procedure

**Figure 1:**
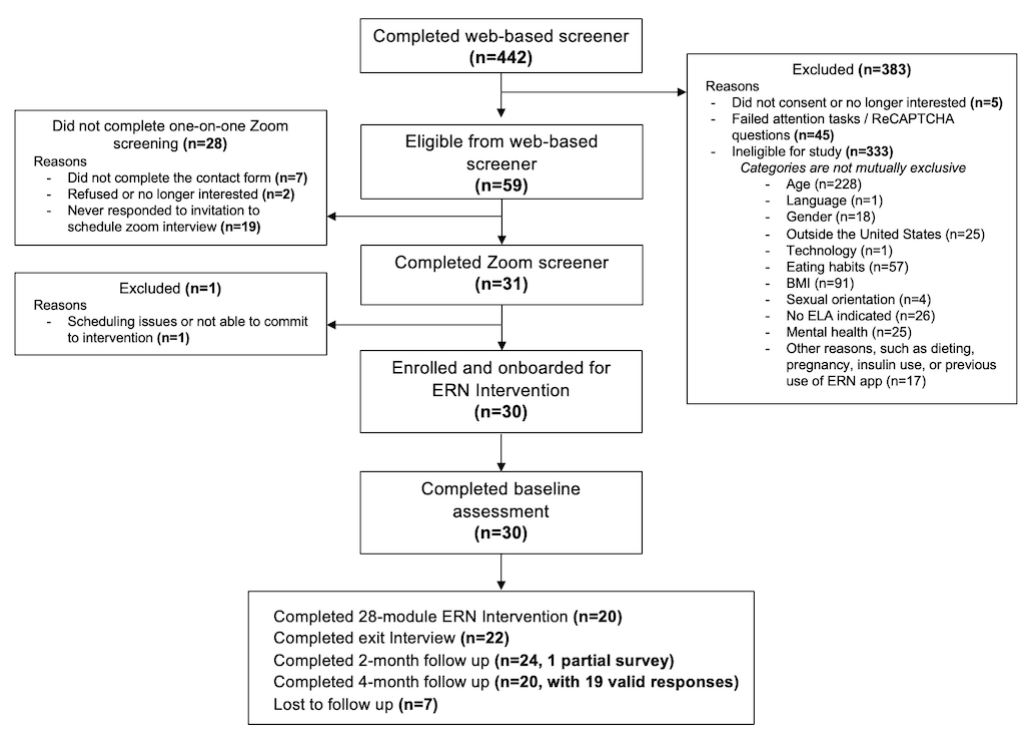
CONSORT (Consolidated Standards of Reporting Trials) flow diagram. ELA: early life adversity; ERN: Eat Right Now.

The study was preregistered as a clinical trial on ClinicalTrials.gov (trial identifier: NCT05201391). Recruitment took place between October and December 2021; we used targeted web-based recruitment by posting on relevant Facebook groups, Reddit threads, and advertisements through the Mindfulness Center at Brown University. Interested individuals completed a brief web-based screening survey to determine initial eligibility, which included the following inclusion criteria: (1) aged 30 to 55 years; (2) BMI ≥25 kg/m^2^; (3) fluent in English; (4) have a smartphone; (5) reside in the United States; (6) experiences food (salty or sweet) cravings and endorses overeating of these foods (ie, responded “yes” to “do you find yourself eating more than you’d like of a particular food or category of foods?”) at least 4 times per week; (7) self-identifies as lesbian, bisexual, queer, or other sexual minority women; and (8) experienced ELA, screened via an adapted measure of childhood abuse for sexual minority individuals [20] that asked if participants experienced forced or nonconsensual sex or sexual contact, physical abuse, and peer victimization. Exclusion criteria were as follows: (1) a current eating disorder; (2) a strict diet (eg, paleo, keto, vegan, or calorie restriction); (3) current use of insulin; (4) pregnancy or trying to become pregnant; (5) previous use of the intervention; (6) a history of a serious mental illness, such as bipolar disorder or psychotic disorders; or (7) imminent risk for suicide. Following initial eligibility screening, a research assistant (RA) contacted potentially eligible participants to schedule a brief Zoom-based (Zoom Video Communications, Inc) screening to go over the study procedures and confirm final eligibility and interest. Participants were asked to show an ID (eg, a driver’s license) on Zoom to verify their age. A total of 442 individuals participated in the web-based screener, and 30 eligible participants were enrolled in the study following the Zoom screen. Figure 1 presents the CONSORT (Consolidated Standards of Reporting Trials) flow diagram including the exclusion and ineligibility of the screened individuals.

Upon completing baseline questionnaires and submitting a weight assessment, an RA welcomed and oriented the participant to the study. During the orientation session, participants were instructed to download the mobile app *Eat Right Now* and provided with a deidentified username and password, and various functions and features of the app were reviewed with them. Participants were instructed to complete the app-based program within 6 to 8 weeks after enrollment.

### Ethics Approval

The study protocol was reviewed and approved by the institutional review board at Brown University (protocol #2106003013).

### Informed Consent and Study Participation

Before participating in the brief web-based screener, interested individuals were asked to review the informed consent form and provide their consent to participate by clicking the “I agree” button. If initially eligible, participants were again provided with information about the study, and their interest and consent were reconfirmed by an RA during the Zoom screen. Each participant was assigned a unique participant ID (PID) that was stored separately from the study data on a secure server. The PID was used for all surveys and for weight data collection. Zoom-based interviews were removed following professional transcription, where any potentially identifiable information was removed from the transcripts, and the PID was used instead. Participants were compensated up to a total of US $120, which included US $30 for the completion of each survey (baseline, after the intervention, and 4-month follow-up) and an additional US $30 for participating in exit interviews. Payments were made through electronically delivered Amazon gift cards.

### Measures

#### Baseline Assessment

At baseline, participants answered questions including demographic information and experience of ELAs, measured using the *Maltreatment Abuse and Exposure Scale* (MAES).

The MAES was used at baseline (T1) to retrospectively assess exposure to various types of childhood maltreatment [21]. Participants answered “Yes/No” to 52 items regarding their childhood adversity experience (eg, “Hit or harmed you so severely that it needed medical attention” in the peer physical bullying subscale). The MAES contains 10 subscales related to experiences of abuse, neglect, and witness of violence. For this study, scores on 8 subscales related to abuse and neglect were calculated due to their proximity to participants’ experiences, including parental physical abuse, parental verbal abuse, parental emotional abuse, peer physical abuse, peer emotional abuse, sexual abuse, emotional neglect, and physical neglect. Higher scores represent a higher amount of ELA in its relevant aspect.

#### Primary and Secondary Outcomes

Primary and secondary outcomes were assessed at baseline (T1, before the intervention), after the intervention (T2; at 2 months), and at follow-up (T3; at 4 months). The primary outcomes of the study included participants’ food cravings, reward-based eating, and weight. The secondary outcomes of the study were mindfulness, depression, anxiety, and emotion regulation.

*Food Craving Questionnaire-Trait, Reduced* was used to assess the experiences of food cravings [22]. Participants rated 15 statements about craving experiences on a 5-point Likert scale ranging from 1 (strongly disagree) to 5 (strongly agree). Participants’ responses were summed such that the total scores could range from 15 to 75. Higher total scores indicated a greater frequency and intensity of food cravings. The Cronbach α values in this study were .92, .89, and .92 at baseline, after the intervention, and at follow-up, respectively.

The *Reward-based Eating Drive Scale* was used to measure eating-related experiences of satiety, preoccupation, and control, which has demonstrated high reliability in samples of women with obesity [23]. Participants rated 13 statements about reward-related eating experiences on a 5-point Likert scale ranging from 0 (*strongly disagree*) to 4 (*strongly agree*). Items were summed such that total scores could range from 0 to 52, and a higher total score indicated a higher reward-based eating drive. The Cronbach α values in this study were .88, .86, and .91 at baseline, after the intervention, and at follow-up, respectively.

#### Weight and Height

Participants were provided a standardized weight scale from Amazon via mail and were asked to record their weight periodically. Specifically, weight data were collected using SMS text message surveys sent via a secure Qualtrics web platform. Participants were reminded to weigh themselves at the same time of the day (eg, morning before breakfast), take a photo on their phone, and upload the photo to the survey link. They were also asked to report their weight via the survey. An RA manually checked the uploaded photos and the participants’ weight to ensure consistency. Height data were collected by self-report at screening and at baseline. Participants’ BMI was calculated using height and weight.

The *Five-Facet Mindfulness Questionnaire* (FFMQ) measures 5 dimensions of dispositional mindfulness [24]: observing (8 items), describing (8 items), acting with awareness (8 items), nonjudging of inner experience (8 items), and nonreactivity of inner experience (7 items). Participants rated 39 statements about dispositional mindfulness on a 5-point Likert scale ranging from 1 (*never or very rarely true*) to 5 (*very often or always true*). A total of 5 subscales were calculated from the sum of their items. A total FFMQ score was calculated from the sum of the 5 subscale scores (possible range 39-195). Higher scores indicate higher levels of mindfulness. The Cronbach α values in this study were .87, .86, and .88 for the FFMQ total at baseline, after the intervention, and at follow-up, respectively.

The *Patient Health Questionnaire-9* measures depression symptom frequency in the past 2 weeks [25,26]. Participants rated 9 items on a 4-point Likert scale from 0 (*not at all*) to 3 (*nearly every day*). Items were summed such that total scores could range from 0 to 21 and higher total scores indicated greater symptom frequency. The Cronbach α values in this study were .86, .82, and .85 at baseline, after the intervention, and at follow-up, respectively.

The *Generalized Anxiety Disorder-7* assesses anxiety symptom frequency in the past 2 weeks [27]. Participants rated 7 items on a 4-point Likert scale from 0 (*not at all*) to 3 (*nearly every day*). Items were summed such that total scores could range from 0 to 21 and higher total scores indicated greater anxiety frequency. The recommended clinical cutoff is ≥8 when determining a probable anxiety disorder [27]. The Cronbach α values in this study were .90, .90, and .94 at baseline, after the intervention, and at follow-up, respectively.

The *Difficulties with Emotion Regulations 18* measure assesses 6 facets of emotion regulation: emotion awareness, emotion clarity, behavior regulation during distress, goal-directed cognition and behavior during distress, acceptance of emotional responses, and coping strategies [28,29]. Participants rated 18 statements on a 5-point Likert scale from 1 (*almost never: 0%-10%*) to 5 (*almost always: 91%-100%).* Total scores could range from 18 to 90, with higher scores indicating greater difficulty with emotion regulation. The Cronbach α values in this study were .94, .93, and .96 for the *Difficulties with Emotion Regulations 18* total at baseline, after the intervention, and at follow-up, respectively.

#### Feasibility and Acceptability

Feasibility and acceptability were assessed after the intervention only via engagement indicators, standard measures, and exit interviews. Feasibility was indicated by attendance to ERN sessions and retention rates. Acceptability was indicated by the evaluation of participants’ satisfaction and perceived usability of the program, measured by the *Client Satisfaction Survey* and the *Adapted System Usability Scale*.

The *Client Satisfaction Survey* consists of 18 items, each rated by participants on a 4-point Likert scale ranging from 1 (*strongly disagree*) to 4 (*strongly agree*). Items assess participant experiences during (eg, “The app program was enjoyable”) and takeaways from (eg, “My eating behaviors have improved since participation in this program”) the mindful eating app program. The total scores could range from 18 to 64, with high scores indicating greater satisfaction. The Cronbach α value was .96 after the intervention.

The *Adapted System Usability Scale* consists of 10 items, each rated by participants on a 5-point Likert scale ranging from 1 (*strongly disagree*) to 5 (*strongly agree*). Items assess the participants’ assessment of app logistics, design, and user friendliness. Participants’ responses were summed such that the total scores could range from 10 to 50. Higher total scores indicate greater perceived system usability. The Cronbach α value was .89 after the intervention.

#### Exit Interview

Feasibility and acceptability were also assessed through an exit interview. All enrolled participants were invited to participate in an exit interview regardless of whether they completed the intervention. Exit interviews focused on participants’ experiences during each phase of the study, including recruitment, onboarding, and intervention. Participants were asked to recall their experience with specific features and components of the intervention program, such as educational videos and mindfulness exercises, as well as their experience applying new skills in their daily life. Exit interviews were conducted via Zoom and lasted 1 to 1.5 hours each.

### Intervention Procedure

We administered an app-based mindful eating intervention aimed at helping people improve their eating habits (described in the study by Taylor et al [30]). The app consists of 28 core modules and 18 “theme weeks” that deliver 3 major components. The first component includes psychoeducational videos (5-15 min/module) designed to teach users to attend to environmental and emotional triggers of eating, types of food that may reinforce craving, and how to apply mindful attention to physiological cues and eating. Participants were able to review educational videos in a designated order using a content-dripping feature (ie, reviewing the videos for module 1 would allow the release of module 2 videos after 24 h). The second component includes brief mindfulness practices (audio recordings, such as body scans and self-compassion) that participants could access for practice. The third component includes tools that help with craving and stress. For example, one of the tools was the “Craving Tool,” wherein users completed a brief mindfulness practice to check in with their body and raise awareness of their feelings. This was followed by feedback about whether these feelings may be consistent with stress, eating and mental habits, or hunger, along with a suggestion to either eat mindfully or use one of the mindfulness practices to cope with cravings. These tools are designed for participants to work with challenges related to unhealthy eating habits and to apply mindfulness in the moment. Participants could customize the settings on the app and their phone for reminders of modules, practice, or built-in tools.

### Analytical Plan

To understand the intervention effects from baseline to after the intervention and follow-up in this exploratory, single-arm trial, we performed 2-tailed, paired *t* tests to compare the results from baseline to after the intervention and follow-up. We also calculated the effect sizes of each outcome using Cohen *d* to quantify the difference. Cohen *d* can be interpreted, according to Cohen [31] conventions, as small (0.20), medium (0.50), and large (0.80). We applied the Benjamini-Hochberg procedure of false discovery rate correction for primary outcomes (weight, food cravings, and reward-based eating) in accordance with established guidelines for adjusting multiple comparisons in randomized controlled trials with multiple primary outcomes [32].

We conducted a post hoc, explorative regression analysis to examine whether certain subpopulations of ELA experience responded better or worse to the mHealth intervention. Specifically, a separate regression analysis was conducted with food cravings or reward-based eating as an outcome variable, whereas different types of ELA were included as a predictor variable while controlling for the corresponding baseline outcome variable. Severity on 8 different types of ELA exposure was measured using the MAES, as described above for measures (eg, parental verbal abuse, parental physical abuse, and emotional neglect). All quantitative data analyses were conducted using R.

Descriptive statistics were used to summarize the feasibility and acceptability surveys. Furthermore, we analyzed qualitative data from the exit interviews using the Framework Method [33], which involved 7 steps. SS, the study principal investigator, who has extensive experience in qualitative research, provided all involved RAs with training on qualitative data coding. First, audio recordings were professionally transcribed. Second, the research team members became familiar with the interviews via the transcripts. Third, 2 researchers engaged in open coding for the first few transcripts to gain a holistic understanding of the data and to inform the development of a more focused framework. Fourth, researchers developed a working analytic framework on feasibility- and acceptability-related data from the interview for focused coding. Fifth, this working analytical framework was applied by indexing all transcripts using the existing categories and codes. Sixth, qualitative data were summarized via charting. Specifically, we used a spreadsheet to generate a matrix. The final step, which was applied throughout the data analysis, involved researchers conducting data interpretation with attention to patterns and connections between categories that emerged from the framework matrix. This was completed via a separate notebook and regular meetings.

## Results

### Reach and Sample Characteristics

A total of 441 individuals completed the web-based screener between October 12, 2021, and December 13, 2021. As shown in Figure 1, age was the most common exclusion reason (228/333, 68.5% were determined to be ineligible). A total of 30 participants were recruited, all of whom completed the baseline assessment.

Table 1 presents the demographic characteristics of the study sample. Participants were aged 40.20 (SD 7.15) years on average. The average BMI of participants at baseline was 36.15 (SD 7.54). Most of the participants were White (28/30, 93%). A majority identified their gender as cisgender (27/30, 90%), whereas a few identified as nonbinary (2/30, 7%) or androgynous (1/30, 3%). Participants identified their sexuality as lesbian (9/30, 30%), bisexual (8/30, 26%), queer (7/30, 23%), pansexual (5/30, 16%), and asexual (1/30, 3%). Most received bachelor’s (9/30, 30%) or graduate (15/20, 50%) degrees and were employed full time (24/30, 80%). Most participants experienced some form of sexual assault in their early life (23/30, 67%). The sample had a wide geographic representation, with 53% (16/30) of participants from states in the Northeast, 23% (7/30) from the Midwest, 17% (5/30) from the South, and 7% (2/30) from states in the West. Although mental health symptoms were not an inclusion criterion, a large proportion of the sample experienced some level of depression (26/30, 87%) or anxiety (24/30, 80%) symptoms at baseline, with the majority in the mild to moderate range of severity.

**Table 1:** Demographic characteristics of the study sample (n=30)^a^.

Demographic characteristic			Participant, n (%)
**Sexual orientation**			
	Lesbian or gay		9 (30)
	Bisexual		8 (26)
	Pansexual		5 (16)
	Queer		7 (23)
	Asexual		1 (3)
**Gender identity**			
	Cisgender person		27 (90)
	Gender nonbinary		2 (6)
	Other (androgynous)		1 (3)
**Race**			
	American Indian or Alaskan Native		1 (3)
	Asian		0 (0)
	Black		0 (0)
	Native Hawaiian and other Pacific Islander		0 (0)
	White		28 (93)
	Multiracial		1 (3)
	Other		0 (0)
**Ethnicity**			
	Hispanic		2 (6)
	Non-Hispanic		28 (93)
**Education**			
	Less than high school		0 (0)
	High school graduate or equivalent		1 (3)
	Some college but no degree		5 (16)
	Bachelor’s or associate’s degree		9 (30)
	Graduate degree		15 (50)
**Employment**			
	Employed full time		24 (80)
	Employed part time		4 (13)
	Homemaker (not looking for a job)		1 (3)
	Retired		1 (3)
**State of residence**			
	Northeast^b^		16 (53)
	Midwest^c^		7 (23)
	South^d^		5 (16)
	West^e^		2 (6)
**Early life adversity**			
	Parental physical abuse		18 (60)
	Physical assault at school		6 (20)
	Missed school due to peer victimization		16 (53)
	**Sexual assault**		23 (76)
		Coerced intercourse	11 (36)
		Unwanted touch	23 (76)
**Depressive symptoms**			
	Minimal		4 (13)
	Mild symptoms		7 (23)
	Moderate symptoms		10 (33)
	Moderately severe		5 (16)
	Severe symptoms		4 (13)
**Anxiety symptoms**			
	No symptoms		6 (20)
	Mild symptoms		10 (33)
	Moderate symptoms		5 (17)
	Severe symptoms		9 (30)

^a^Mean age was 40.20 (SD 7.15) years. Mean BMI was 36.15 (SD 7.54) kg/m^2^.

^b^The Northeast region includes Connecticut, Delaware, District of Columbia, Maine, New Hampshire, New Jersey, New York, Maryland, Massachusetts, Pennsylvania, Rhode Island, and Vermont.

^c^The Midwest region includes Illinois, Indiana, Iowa, Kansas, Nebraska, North Dakota, Ohio, Michigan, Minnesota, Missouri, South Dakota, and Wisconsin.

^d^The South region includes Alabama, Arkansas, Florida, Georgia, Kentucky, Louisiana, North Carolina, Oklahoma, Missouri, South Carolina, Tennessee, Texas, Virginia, and West Virginia.

^e^The West region includes Alaska, Arizona, California, Colorado, Hawaii, Idaho, Montana, Nevada, Oregon, Utah, Washington, and Wyoming.

### Baseline to Postintervention and Follow-Up Effect

#### Primary Outcomes

**Figure 2:**
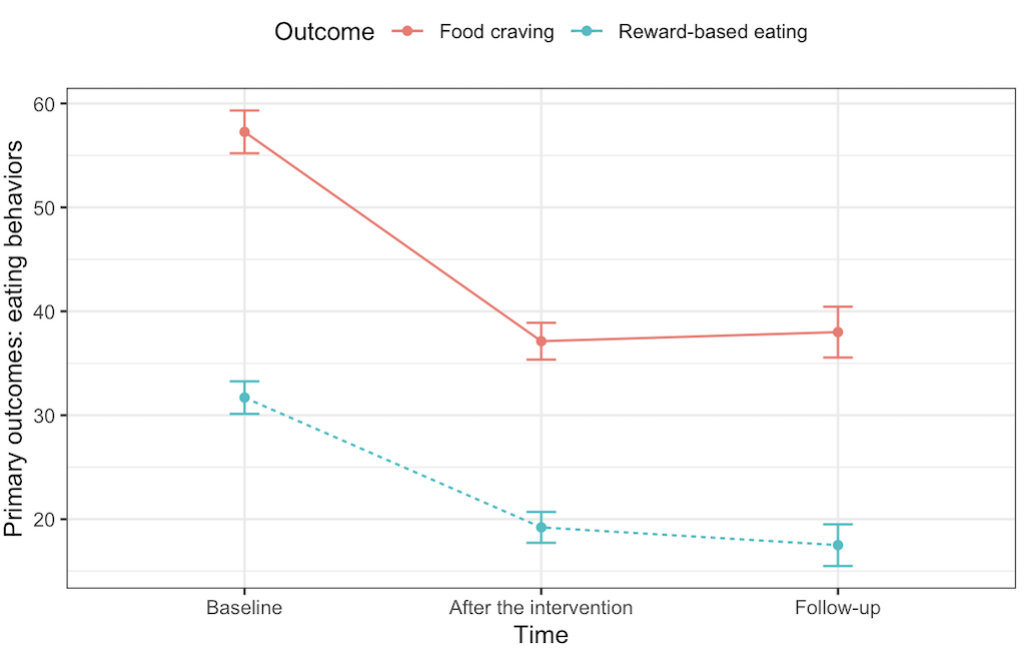
The effect of the Eat Right Now mobile app from baseline to after the intervention and follow-up on eating behavior changes among sexual minority women with early life adversity.

**Figure 3:**
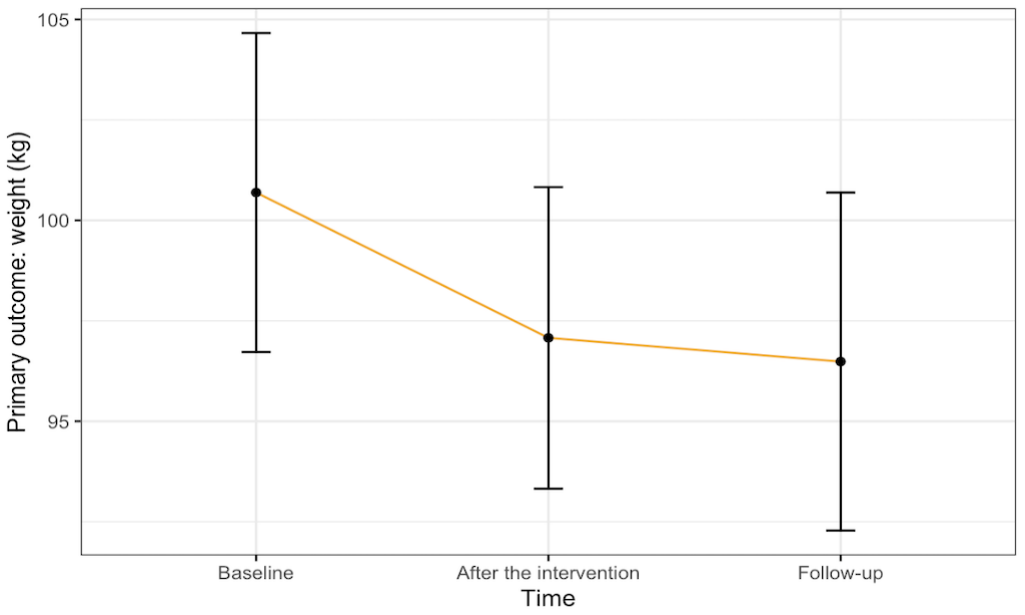
The effect of the Eat Right Now mobile app from baseline to after the intervention and follow-up on weight among sexual minority women with early life adversity.

Table 2 presents the effect of the mindfulness app on participants from baseline (T1) to after the intervention (T2; 2 months) and at follow-up (T3; 4 months). On average, the participants lost 4.21 kg (4.2% body weight) during the follow-up assessment. Weight loss was slightly higher among those with a larger BMI (>30 kg/m^2^; n=24), with an average of 4.97 kg (4.7% body weight). There were significant and large reductions in food cravings from baseline to after the intervention (Cohen *d*=1.76; *P*<.001). This large reduction effect persisted until the 4-month follow-up (Cohen *d*=1.64; *P*<.001). Reward-based eating also decreased after the intervention, with a large effect size (Cohen *d*=1.42; *P*<.001) and continued to decrease at follow-up (Cohen *d*=1.56; *P*<.001). The effects on food cravings and reward-based eating at both time points remained significant after applying for a false discovery rate correction (*P*<.001). The effects of the program on the primary outcomes are illustrated in Figures 2 and 3.

**Table 2:** Baseline to after the intervention and follow-up changes in primary and secondary outcomes.

Variables			Baseline, mean (SD)		After the intervention, mean (SD)		Follow-up, mean (SD)		Baseline to immediate after the intervention, Cohen *d*		Comparison between baseline and after the intervention				Baseline to follow-up, Cohen *d*		Comparison between baseline and follow-up		
											*t* test (*df*)		*P* value^a^				*t* test (*df*)		*P* value^a^
**Primary outcomes**																			
	Weight	100.69 (21.74)		97.08 (18)		96.48 (18.81)		0.18		0.62 (22)		.54		0.20		0.46 (19)		.65	
	Food craving	57.27 (11.27)		37.12 (8.69)		38.00 (10.94)		1.76		7.32 (23)		*<.001* ^b^		1.64		6.51 (19)		*<.001*	
	Reward-based eating	31.7 (8.57)		19.21 (7.28)		17.5 (8.98)		1.42		6.19 (23)		*<.001*		1.56		6.71 (19)		*<.001*	
**Secondary outcomes**																			
	Mindfulness	118 (28.4)		134.21 (25.92)		134.75 (32.05)		0.51		−4.86 (23)		*<.001*		0.49		−3.21 (19)		.005	
	Depression	11.67 (6.26)		6.38 (4.57)		6.2 (4.55)		0.89		5.57 (23)		*<.001*		0.98		4.46 (19)		*<.001*	
	Anxiety	9.9 (5.52)		6.33 (4.86)		7.15 (5.97)		0.63		4.47 (23)		*<.001*		0.50		2.89 (19)		.009	
	Emotion dysregulation	46.03 (15.23)		38.29 (12.48)		37.15 (16.05)		0.44		3.54 (23)		.002		0.44		2.64 (19)		.02	

^a^The *P* values of food craving and reward-based eating after the intervention and at follow-up remained significant at the <.001 level after false discovery rate correction.

^b^Italicization indicates significance.

#### Secondary Outcomes

The mindfulness app was effective in improving mindfulness, depression, and anxiety symptoms and emotional dysregulation. The app had moderate effects on mindfulness (Cohen *d*=0.51 after the intervention; *P*<.001 and Cohen *d*=0.49 at follow-up; *P*=.005), anxiety (Cohen *d*=0.63 after the intervention; *P*<.001 and Cohen *d*=0.50 at follow-up; *P*=.009), and emotion dysregulation (Cohen *d*=0.44 after the intervention; *P*=.002 and Cohen *d*=0.44 at follow-up; *P*=.02). Reduction in depressive symptoms was large after the intervention (Cohen *d*=0.89; *P*<.001) and the effect increased slightly at follow-up (Cohen *d*=0.98, *P*<.001).

### Explorative Analysis on ELA Characteristics Associated With Intervention Outcomes

Post hoc, explorative analysis (Table 3) examined the association between ELA experience and reduction in food cravings and reward-based eating from baseline to the 4-month follow-up. Those with higher levels of parental verbal abuse (B=−3.79, 95% CI −7.24 to −0.34; *P*=.03) and parental emotional abuse (B=−3.56, 95% CI −6.29 to −0.83; *P*=.01) had greater reductions in food craving. Participants who reported higher parental emotional abuse also experienced greater reductions in reward-based eating (B=−2.59, 95% CI −4.80 to −0.38; *P*=.02).

**Table 3:** Explorative analysis on baseline early life adversity characteristics and intervention outcome.

Baseline variables			Food craving (T3^a^)					Reward-based eating (T3)		
			B (95% CI)		*P* value		B (95% CI)			*P* value
**Dimension of abuse**										
	Parental physical abuse	−0.83 (−3.30 to 1.64)		.49		−0.67 (−2.59 to 1.25)			.47	
	Parental verbal abuse	−3.79 (−7.24 to −0.34)		.03		−1.28 (−4.32 to 1.77)			.39	
	Parental emotional abuse	−3.56 (−6.29 to −0.83)		.01		−2.59 (−4.80 to −0.38)			.02	
	Peer physical abuse	−1.36 (−4.60 to 1.87)		.39		−0.11 (−2.72 to 2.49)			.93	
	Peer emotional abuse	0.90 (−1.89 to 3.69)		.51		0.44 (−1.77 to 2.64)			.68	
	Sexual abuse^b^	−1.31 (−3.81 to 1.19)		.28		0.02 (−2.04 to 2.07)			.99	
**Dimension of neglect**										
	Emotional neglect	0.09 (−0.15 to 0.33)		.43		0.07 (−0.12 to 0.25)			.46	
	Physical neglect	−3.32 (−6.92 to 0.28)		.07		−1.62 (−4.67 to 1.42)			.28	

^a^T3: 4-month follow-up.

^b^Sexual abuse included experiences from both within and outside of one’s household. The outcome variable (food cravings or reward-based eating) at baseline was controlled for in all regression models.

### Quantitative Indicators of Feasibility and Acceptability

#### Intervention Feasibility

Engagement and attrition were examined as indicators of the feasibility of the intervention. Among the 30 enrolled participants, 25 (83%) were retained after 2 months (T2) and 20 (67%) were retained at the 4-month follow-up (T3). A total of 20 (67%) participants completed all 28 core modules of the program. Among those who did not complete the full 28 modules (n=10), the average completion was 8.6 (range 0-25) modules.

#### Acceptability

Intervention acceptability was assessed via both quantitative measures and qualitative exit interviews. As shown in Table 4, participants’ responses were on the positive spectrum regarding satisfaction (>2.5) and usability (>3 on items indicating usability or <3 for items with reverse meaning). Ratings on some program satisfaction items were lower than those of others, especially those regarding participants’ improvement in emotion regulation, self-acceptance, and stress coping, as well as whether the presentation of content inspired the interest for learning. No adverse events or adverse effects were observed.

**Table 4:** Feasibility and acceptability of the mindfulness mobile health app program.

				Values
**Program satisfaction^a^, mean (SD)**				
	Total satisfaction			54.28 (9.73)
	**Evaluation by item**			
		1. I learned a lot from this app.	3.24 (0.72)	
		2. I will be able to apply what I learned from this intervention app in my life.	3.24 (0.66)	
		3. I was able to do the activities.	3.08 (0.76)	
		4. The intervention app was well organized.	3.16 (0.75)	
		5. The topics in the intervention program were interesting.	3.24 (0.66)	
		6. The presentation of the app stimulated my interest in the material.	2.80 (0.82)	
		7. The topics of the intervention app were relevant to my life.	3.28 (0.68)	
		8. The app program was enjoyable.	3.00 (0.65)	
		9. I would recommend this app program to others.	3.16 (0.62)	
		10. I felt comfortable during the intervention app program.	3.28 (0.54)	
		11. My eating behaviors have improved since participation in this program.	2.80 (0.65)	
		12. My lifestyle behaviors have improved since participation of this program.	2.84 (0.69)	
		13. I have learned how to regulate my emotions through this program.	2.64 (0.64)	
		14. I have become more accepting of myself through this program.	2.84 (0.75)	
		15. I have learned how to cope with stress through this program.	2.76 (0.60)	
		16. I have learned how to better care for myself through this program.	2.84 (0.69)	
		17. I can cope with food-related cravings better now.	3.08 (0.70)	
		18. I have a healthier relationship with food through this program.	3.00 (0.71)	
**Program usability^b^, mean (SD)**				
	Total usability			43.16 (6.05)
	**Evaluation by item**			
		1. I think that I would like to use the ERN^c^ mobile app frequently.	3.60 (0.96)	
		2. I found the ERN app unnecessarily complex.	1.72 (0.94)	
		3. I thought the ERN app was easy to use.	4.16 (0.99)	
		4. I think that I would need the support of a technical person to be able to use the ERN app.	1.00 (0.00)	
		5. I found the various functions in the ERN app were well integrated.	3.80 (1.04)	
		6. I thought there was too much inconsistency in the ERN app.	1.48 (0.82)	
		7. I would imagine that most people would learn to use the ERN app very quickly.	4.48 (0.65)	
		8. I found the ERN app very cumbersome (awkward) to use.	1.96 (1.24)	
		9. I felt very confident using the ERN app.	4.56 (0.77)	
		10. I needed to learn a lot of things before I could get going with the ERN app.	1.28 (0.61)	
**Adverse effect (yes or no), n (%)**				
	Has the program caused any negative impact in your life?			0 (0)

^a^Item range: strongly disagree (score=1) to strongly agree (score=4).

^b^Item range: strongly disagree (score=1) to strongly agree (score=5).

^c^ERN: Eat Right Now.

### Qualitative Findings on Intervention Feasibility, Acceptability, and Experienced Impact

Multimedia Appendix 1 presents the findings of the exit interviews (n=22). Three key themes emerged, specific to app acceptability and barriers to engagement, usability of app features, and experienced impact.

### Key Theme 1: Program Acceptability and Engagement Barriers

#### The Program Aligned With Goals and Expectations

Participants expressed that they came to the program hoping to reframe their relationship with eating and their weight (eg, to decrease self-judgment; for some, to lose weight; and for better health) as well as for mindfulness training:

Just to get on track and figure out how I can use this mindfulness tool to jumpstart my weight loss and work toward my health goals. I know what to do, and I know I need to stick with a program, and I thought that the app would be a good choice.

PID 2411, 41-year-old bisexual woman

Try to be kind to yourself and know that being fat is not a bad thing. My goal was not to lose weight. It was just to see if anything would change. I know I do binge eat and I do eat a lot of sweets and I’m an emotional-response eater. I was curious about how my behaviors would change in the program.

PID 9825, 32-year-old queer woman

They also expected to apply mindfulness practices to change their relationship with eating and food in a direction conducive to health (eg, lower eating to cope, emotional eating, and healthier choices) as well as, in some cases, lose weight:

I think it pretty much lined up with my expectations, particularly when I understood a little bit more about what it was about and started using the app. I think it pretty much lined up with what I expected.

PID 9825, 32-year-old queer woman

I’ve done a lot of stuff with mindfulness in terms of mental health but applying it to eating was a whole new world. It’s really cool, but you can do the same thing but with eating. I was hoping to see if something like this would be helpful and change the way I think about food and deal with weight.

PID 5346, 34-year-old lesbian genderqueer individual

It was on mindfulness and eating, learning about how it can relate and help in eating issues, cravings, and things like that.

PID 2411, 41-year-old bisexual woman

Almost all participants indicated that the app met expectations and exceeded a few. Several appreciated that they felt it had no shame-inducing language or content specifically mentioning weight. Some explained that there was a learning curve, and one stated that they found it less appealing than other meditation apps:

I feel like it could be more modern or more appealing. It looks like a well-designed app but like older, in that the design isn’t super modern.

PID 7522, 38-year-old lesbian woman

#### The Program Helped Meet Health Goals but Could Be Improved Through Individual Support and Body Positive Language

Participants overwhelmingly indicated satisfaction with the content and that it helped them move toward their specific health and well-being goals. The program helped accomplish goals related to food (eg, reframing eating, working with craving, savoring food, and healthier choices), learning mindfulness (eg, specific practices, application in eating, and relearning practices they’d forgotten), and increasing self-care or kindness (eg, silencing internalized oppression and decreasing self-judgment). However, several participants indicated that they would have liked an individual support component and education on dietary choices:

It was totally relevant. I feel like that’s what it is all about like breaking habit loops and trying to get at the things underneath of why you’re eating. Not demonizing like “Oh well, you just need to like restrict calories, you need to have more willpower and just stick to this and drive yourself harder,” but it was about trying to understand like what’s going on inside.

PID 8587, 38-year-old bisexual woman

In addition, several participants believed that the app could be improved by addressing self-kindness and the relationship with their body directly by creating content that celebrates diverse body types (ie, body positivity). Specifically, they believed that the program should prioritize content focused on fat is beautiful, “fat is not a bad thing” (PID 9825), reducing the focus on weight reduction as a goal and educating on how the body changes as people age:

To change people’s perspective about sizes because people get hung up on numbers. Unfortunately, this still does some numbering. Like, with weight numbers, but the number is the same for everyone, so the, the number of 185 looks different on so many different people. And so, if there was a way to kind of incorporate how they’re just numbers, they’re not really defining anything else.

PID 4671, 41-year-bisexual woman

#### Barriers to Engagement: Time, Technical Difficulties, and Lack of Relatability

Barriers to program engagement can be divided into 3 categories: lack of time, technical difficulties, and the limited relatability of a cisgender White male teacher. Concerning time, participants reported that contracting COVID-19, childcare, holidays (Thanksgiving and Christmas), and overall busy schedules limited their ability to engage. Technical difficulties were described as glitches in the app, such as repeating education modules and difficulty navigating modules:

I think just really busy schedule. And my schedule changes every day so it’s not like I have set days or set times every day that I know I’m going to have flexible. So, I think it’s a lot of just like trying to schedule.

PID 8710, 31-year-old lesbian woman

I had a couple of issues with the videos where on a couple of particular days it didn’t seem to go to the next one. I’d open the app the next day and it would still say the previous day’s ones. I tried to figure out what I did, if I didn’t close it outright or if it didn’t finish all the way to the very end. I let them play all the way through.

PID 4070, 44-year-old queer woman

The strongest feedback was from 5 participants regarding the lack of relatability of a White male facilitator. Participants felt that a cisgender White male teacher was not representative of not just their sexuality and gender but also the range of body types of those using the app. Some participants indicated that although the teacher seemed kind, they felt that they could not relate to the issues they would face in their daily life:

That was like a little challenging. I wish there were different people, a variety of people who had all different kinds of bodies, and were just more reflective of what that the people using the app might look like and sound like and be like. Or if those perspectives could be incorporated in some way.

PID 8587, 38-year-old bisexual woman

I think, also, it’s hard for me as a queer woman to relate to the guy who’s like teaching the modules as they are an older, fit person. It was very engaging and there’s nothing wrong ... I don’t have any criticisms of him, but I think there’s something in my head that was stopping me from fully getting into it and relating. There was kind of a bit of disconnect and a bit of suspicion, and I guess that held me back a little bit.

PID 7522, 38-year-old lesbian woman

### Key Theme 2: Usability of App Program Features

#### Overall Program Ease of Use

Almost all participants stated that they found the program easy to use and navigate and liked the various features of the app (eg, separate web-based community area, features menu, and ability to track progress). Most participants felt that the program was appealing, that the program had a clean look, and that the content was presented in a user-friendly manner. Overall, participants believed that the content was presented well using descriptions such as “user-friendly,” “comfortable,” “intuitive,” and “easy to navigate.” However, 2 participants disagreed that they found it unappealing, confusing, and “clunky”:

It was user-friendly. It was easy to find things. I liked that the dashboard had like this part, the check-in, the craving tool, like what module I’m doing today and all the things I’m using regularly. It’s all there when I open the app.

PID 4020, 33-year-old bisexual woman

I thought the app was very ugly and it was not very user friendly, and it felt like it was just difficult like it was clunky. It was just like it wasn’t very intuitive.

PID 9825, 32-year-old queer woman

#### The Education Modules Were Understandable, Useful, and of Good Length

The education modules are the core intervention component and are the feature most engaged with by the participants. Participants explained that they worked well because they could be played while multitasking (eg, driving), helped increase mindful eating, had useful scientific explanations, and fit with their existing knowledge and behaviors. Participants reflected that the modules were of a good, “bite-sized” length (ie, 2-5 min each, approximately 3-5 videos per module).

#### The Program Tools Were Commonly Unused, and Many Found Them Unclear

The program also consists of supportive ecologic tools (eg, stress test, general check-in, and craving tool) that are accessed through both push notifications and as needed by participants. Of these tools, 1 participant reported using the most, and found to be the most useful, was the general check-in tool. However, only half of the participants described accessing the tools, and only 2 participants reported using almost all of them regularly. Others described them as unclear, confusing, not needed, and “a nuisance.” A few participants reported that although they did not use the features regularly, they found them helpful when they did, specifically the craving tool and check-in features:

I liked the throughout-the-day check-ins. Like it wasn’t like okay, so I listened to the app and then go on with my day and forgot about it for the rest of the day. I would listen to it and then it would check on me. It would at least remind me, even if I didn’t do every single check in, it would remind me like this is what you’re working for right now, keep going.

PID 4020, 33-year-old bisexual woman

#### Mindfulness Exercises Were Used Regularly but Could Be Improved With Diverse Teachers

Among the interviewed participants, only 4 reported not engaging in mindfulness practices, at least daily. The body scan and loving-kindness practices were the meditations that participants reported using most often. The loving-kindness practice helped improve self-care and increase kindness toward people in their lives. The body scan allowed several participants to develop an awareness of their bodies. Participants noted that mindfulness exercises could be improved if they were offered by different teachers (eg, racial and ethnic minority women) rather than a male voice. Several participants did not enjoy longer practices or repetitiveness of different lengths of practice:

Body scan is fabulous. The love and kindness is excellent.

PID 5762, 47-year-old bisexual woman

I mentioned earlier, just the guy like presenting it just being the fit older white guys just ... you know it didn’t draw me, so maybe somehow having it be more inclusive, in a way, would be good.

PID 7522, 38-year-old lesbian woman

### Key Theme 3: Integration and Changes Experienced After Using the App Program

#### Changes in Eating, Eating Behaviors, and Weight Because of Program Use

Participants reported experiencing changes in a variety of areas in their lives that they attributed to the program. Eating and improvements in their relationship with food was the most endorsed. They explained that they experienced a higher capacity to manage binge eating, food craving, and be present while eating:

Before I started using the app, I wouldn’t really think about it. I’d pause whatever I was doing, and I would go into the kitchen and just look for food without any real thought process, I want to eat. The app forced me to…Say I was watching a show, I like to binge-watch shows. I would pause the show and I’d be like EAT, well wait why? Well, why eat? And then be like okay, bored more so than hungry.

PID 5346, 34-year-old lesbian genderqueer individual

Some also detailed the lifestyle changes they had made because of the program, including meal prepping, healthier buying behaviors, substitution, and cooking healthier holiday meals. Some participants noted losing weight as well as reframing their relationship with weight and body toward a more caring, nonshaming approach:

Simply just to slow down and really think about why I’m eating, if I’m really hungry or thirsty, if I just need to ride out the cravings. Having a bite or two of ice cream instead of a bowl is fine. Or if I’m craving sweets, I can have an apple. Those tips that came through on the app were really helpful.

PID 2411, 41-year-old bisexual woman

I think the other piece that was really big was like getting in touch with the feelings of discomfort that come with overeating like that disenchantment part which is so like logical like duh. I don’t think I had really spent time feeling what that’s physically like in my body versus just like guilt or shame or judgment.

PID 8587, 38-year-old bisexual woman

#### Improvements in Mental Health and Well-Being by Completing the App Program

Many participants noticed changes in their mental health because of the program while a few did not. Of those who described such changes, they talked about how using the program motivated them to engage in self-care, reduce self-judgment and shaming, and improve their awareness and self-confidence. In addition, the skills they learned allowed them to ground themselves and pause using mindfulness practices (eg, Recognize, Allow, Investigate, and Note and breathing) to decrease anxiety, stress, and cravings:

Yeah, I think the app gave me my own space to work on myself and my health. That’s something I really needed—a sacred space for myself and my mental health—my own space to continue on the things that I need to do for myself.

PID 2794, 52-year-old pansexual, androgenous individual

It definitely helped me kind of boost my confidence, a little bit and but I’m not sure if that’s related to you know me being queer or just overall.

PID 9658, 49-year-old bisexual woman

It helped me with my thoughts and my anxiety. It like helps and the breathing techniques and stuff like that helps me and it helps me focus on myself and what was going on with myself.

PID 2517, 41-year-old bisexual woman

## Discussion

### Principal Findings

This early phase feasibility trial with middle-aged sexual minority women who experienced ELA and were at risk of obesity represents an initial effort to understand the use of a mindfulness-based mHealth approach that engages this underserved population and improves health outcomes. The findings provide initial support for remote recruitment and an app-delivered mindfulness intervention with sexual minority women. As the first study to contribute to mHealth intervention research on eating behaviors with sexual minority women, this fills an important gap in the literature regarding the use of mHealth for health promotion in this population. In addition, it highlights the need to address both eating behaviors and mental health concerns and use an app-based delivery model as an engagement approach with this marginalized population.

It has been well documented that sexual minority women underuse health services and are less satisfied with the health system compared with their heterosexual female peers [34]. Anticipation of stigma and bias in interactions with the health system related to being LGBTQ+ and overweight may further prohibit them from seeking professional assistance. Thus, we used a remote and internet-based strategy for all phases of the study, including recruitment, enrollment, intervention delivery, and assessment. This allowed us to capitalize on resources such as social media and web-based LGBTQ+ groups (eg, Facebook), as well as overcome geographic limitations and stigma-related barriers. Internet-based approaches may also fit well with this population, as research suggests that LGBTQ+ individuals spend more time using the internet to seek health information than their heterosexual peers [35]. We were able to complete recruitment within 2 months, and the recruitment or study flow suggests a high demand and potential need to conduct a larger clinical trial with a wider age range to fully address the health needs of adult sexual minority women in the future.

Engagement is a critical issue in mHealth interventions. A recent meta-analysis on mHealth-based mental health intervention studies showed that the proportion of participants who completed all the modules or sessions was 34% to 36% for anxiety- and depression-related trials and 64% for general mental health [36]. For trials that took place entirely on the web, retention rates averaged 56.6% [36]. Findings from the current trial showed overall satisfactory engagement (66.7% completed all modules) and retention (83.3% and 66.7% after the intervention and at the 4-month follow-up, respectively). However, several areas of improvement were identified. Participants particularly noted the importance of having diverse teachers represented in the modules and diverse voices on mindfulness recordings to help them relate to and engage more actively in the program. Other adaptation suggestions for improving engagement include shorter practices, individualized support to enhance motivation and address mental health needs, and further endorsement of body positivity. All these points point to the importance of tailoring the program to this population. Notably, many participants did not endorse losing weight as a goal and shared their values in body shape diversity and body positivity (eg, “fat is beautiful”), which may also explain the small effect size in weight change. As body positivity is a highly relevant cultural value among sexual minority women and can serve as a protective factor against weight stigma [37-40], endorsing language and examples consistent with this value is important for enhancing engagement. Nonetheless, a 4.2% weight loss could still produce health benefits (eg, improvement in blood sugar and blood cholesterol) [41]. The average 4.21 kg weight loss in this sample was considerably higher than the average weight loss of 2.25 kg found in a meta-analysis of mHealth randomized controlled trials [42], although such a difference in size is likely due to the lack of a comparison group in this study.

It is worth noting that although psychological distress was not an inclusion criterion, 86.7% of the sample had at least mild depressive or anxiety symptoms at baseline. Meta-analyses have revealed strong correlations of food addiction with depression (*r*=0.46) and anxiety (*r*=0.48) [43], and mental health concerns were also elevated among sexual minority women and those who experienced childhood adversity [6,8]. Thus, integrating psychological health into programs aiming to address eating behavior in this population can be particularly relevant. Although our mindful eating program does not specifically target mental health symptoms, mindfulness as a skill has been shown to improve depressive and anxiety symptoms [44] and its effect was also observed among participants from qualitative findings. Exploratory analysis suggested that those who experienced higher parental verbal and emotional abuse had higher reductions in food craving and reward-based eating. This finding is consistent with prior research suggesting that individuals who have experienced ELA may be particularly responsive to mindfulness interventions [19,45]. As parental abuse affects eating behavior through heightened emotional reactivity and emotional eating as a coping mechanism (eg, numbing one’s emotions via eating) [46], as suggested by qualitative findings (theme 3 regarding the effect on mental health and well-being), mindfulness practice may be particularly relevant for this subpopulation by disrupting the pathway from abuse to food craving.

### Study Limitations and Directions for Future Research

There are several major limitations to this study. First, although the study had a wide geographical reach, most of the sexual minority women recruited were White (28/30, 93%) and well educated (24/30, 80% had a college degree or above). As obesity also disproportionately affects sexual minority racial and ethnic minority women [6], future research should form strategies to recruit a racially diverse sample of sexual minority women. Second, although the feasibility of this app may be considered satisfactory compared with average mHealth programs [36], the single-arm design makes it difficult to ascertain how this intervention performs in terms of feasibility, engagement, and acceptability compared with an active control. In a related vein, lack of control limits our understanding of the intervention’s efficacy. Third, given the small sample size, we were not able to fully examine the mechanisms through which mindfulness may improve eating behavior in this population (eg, by reducing psychological distress and emotion dysregulation). The finding related to which individuals experienced the most success with this intervention (ie, those with higher verbal or emotional abuse) also needs to be confirmed via a larger trial.

### Conclusions

Sexual minority women are disproportionately more likely to be burdened by obesity as well as ELA, which can contribute to psychological distress and unhealthy eating, yet there has been a dearth of intervention research with this population. A mindfulness-based mHealth approach may offer benefits in addressing unhealthy eating habits and promoting weight loss and psychological health among sexual minority women. The results of this study provide the proof-of-concept that warrants intervention adaptation (eg, with more diverse teacher representation, add-on of individual check-ins) and further evaluation through a larger randomized controlled trial. If proven to be efficacious, and if it is able to be successfully implemented in the real world, a mindfulness-based mHealth approach could be an inexpensive and scalable method that could be widely disseminated to reduce the public health burden of obesity and unhealthy eating among sexual minority women.

## Appendix

Multimedia Appendix 1 Themes of qualitative analysis.

## Abbreviations

CONSORT: Consolidated Standards of Reporting Trials
ELA: early life adversity
FFMQ: Five-Facet Mindfulness Questionnaire
LGBTQ+: lesbian, gay, bisexual, transgender, and queer
MAES: Maltreatment Abuse and Exposure Scale
PID: participant ID
RA: research assistant

## Data Availability

The data sets generated and analyzed during this study are not publicly available to protect participant privacy but are available from the corresponding author upon reasonable request.
