# Supplementary material for: Mindfulness-Based Mobile Health to Address Unhealthy Eating Among Middle-Aged Sexual Minority Women With Early Life Adversity: Mixed Methods Feasibility Trial
Source: J Med Internet Res. 2023 Sep 26;25:e46310. doi: 10.2196/46310 (PMC10565623; doi:10.2196/46310)
Supplement: Multimedia Appendix 1 [file jmir_v25i1e46310_app1.docx]

**Supplementary Table 1**. Themes of qualitative analysis

| **Themes & Sub-Themes** | **Code** |  | **Representative Quote** |
| --- | --- | --- | --- |
| **Key Theme 1: Program acceptability and engagement barriers** | | | |
| Program aligned with goals and expectations. | *Goals for the study* |  | “Just to get on track and figure out how I can use this mindfulness tool to jumpstart my weight loss and work toward my health goals. I know what to do, and I know I need to stick with a program, and I thought that the app would be a good choice." (2411, 41 y/o, bisexual, female)  “Try to be kind to yourself and know that being fat is not a bad thing. My goal was not to lose weight. It was just to see if anything would change. I know I do binge eat and I do eat a lot of sweets and I’m an emotional-response eater. I was curious about how my behaviors would change in the program.” (9825, 32 y/o, queer, female) |
|  | *Reasons for joining* |  | “I’ve done a lot of stuff with mindfulness in terms of mental health but applying it to eating was a whole new world. It’s really cool but you can do the same thing but with eating. I was hoping to see if something like this would be helpful and change the way I think about food and deal with weight.” (5346, 34 y/o, lesbian, genderqueer) |
|  | *Understanding of the study*  *Content relevance and helpfulness*  *App impressions* |  | “It was on mindfulness and eating, learning about how it can relate and help in eating issues, cravings, and things like that.” (2411)  “I think it pretty much lined up with my expectations, particularly when I understood a little bit more about what it was about and started using the app. I think it pretty much lined up with what I expected.” (9825, 32 y/o, queer, female)  "I feel like it could be more modern or more appealing. It looks like a well-designed app but like older, in that the design isn't super modern." (7522, 38 y/o, lesbian, female) |
| Program helped meet health goals but could be improved through individual support and body positive language. | *Content relevance and helpfulness*  *Expected but did not get from the app*  *Aspects of the app that did not help* |  | "It was totally relevant. I feel like that's what it is all about like breaking habit loops and trying to get at the things underneath of why you're eating. Not demonizing like ‘Oh well, you just need to like restrict calories, you need to have more willpower and just stick to this and drive yourself harder’, but it was about trying to understand like what's going on inside.” (8587, 38 y/o, bisexual female)  “To change people's perspective about sizes because people get hung up on numbers. Unfortunately, this still does some numbering. Like, with weight numbers, but the number is the same for everyone, so the, the number of 185 looks different on so many different people. And so, if there was a way to kind of incorporate how they're just numbers, they're not really defining anything else.” (4671, 41 y/o, bisexual, female) |
| Barriers to engagement: time, technical difficulties, and lack of relatability. | *Barriers to engagement* |  | “I think just really busy schedule. And my schedule changes every day so it's not like I have set days or set times every day that I know I’m going to have flexible. So, I think it's a lot of just like trying to schedule.” (8710, 31 y/o, lesbian, female)  “I had a couple of issues with the videos where on a couple of particular days it didn’t seem to go to the next one. I’d open the app the next day and it would still say the previous day's ones. I tried to figure out what I did, if I didn’t close it outright or if it didn’t finish all the way to the very end. I let them play all the way through.” (4070, 44 y/o, queer, female)  “That was like a little challenging. I wish there were different people, a variety of people who had all different kinds of bodies and were just more reflective of what that the people using the app might look like and sound like and be like. Or if those perspectives could be incorporated in some way." (8587, 38 y/o, bisexual, female)  "I think, also, it's hard for me as a queer woman to relate to the guy who's like teaching the modules as they are an older, fit person. It was very engaging and there’s nothing wrong … I don't have any criticisms of him, but I think there’s something in my head that was stopping me from fully getting into it and relating. There was kind of a bit of disconnect and a bit of suspicion, and I guess that held me back a little bit." (7522, 38 y/o, lesbian, female) |
| **Key Theme 2:** **Usability of app program features** | | | |
| Ease of use, look and feel of the app program overall. | *App look & feel*  *User-friendliness*  *App logistics* |  | “It was user-friendly. It was easy to find things. I liked that the dashboard had like this part, the check-in, the craving tool, like what module I’m doing today and all the things I’m using regularly. It’s all there when I open the app.” (4020, 33 y/o, bisexual, female)  “I thought the app was very ugly and it was not very user friendly, and it felt like it was just difficult like it was clunky. It was just like it wasn't very intuitive.” (9825, 32 y/o, queer, female) |
| Education modules overall were understandable, useful, and good length. | *Experience of modules overall*  *Unhelpful parts of modules*  *What Worked Well* |  | “The videos and they were great. like I said they were very consistent, the auto-play was free, so I didn't have the chance to pause and go do something else, it automatically led me into the next part of it. Yeah, those were great.” (5762, 47 y/o, bisexual, female) |
| Program tools were useful when used for some, but many found them unclear. | *App tools experience*  *What Worked Well* |  | “I used the check in a few times but I didn’t really see use from it so I didn't use it. I think I used the craving tool one time, again I didn’t find it useful.” (5320, 30 y/o, bisexual, female)  “I liked the throughout-the-day check-ins. Like it wasn’t like okay, so I listened to the app and then go on with my day and forgot about it for the rest of the day. I would listen to it and then it would check on me. It would at least remind me, even if I didn’t do every single check in, it would remind me like this is what you’re working for right now, keep going.” (4020, 33 y/o, bisexual, female) |
| Mindfulness exercises were used regularly but could be improved with diver teachers. | *Mindfulness practices frequency*  *Mindfulness practices helpful*  *Mindfulness practices unhelpful* |  | “Body scan is fabulous. The love and kindness is excellent.” (5762, 47 y/o, bisexual, female)  "I mentioned earlier, just the guy like presenting it just being the fit older white guys just… you know it didn’t draw me, so maybe somehow having it be more inclusive, in a way, would be good." (7522, 38 y/o, lesbian, female) |
| **Key Theme 3: Integration and Changes Experienced after Using the App Program** | | | |
| Changes in eating, eating behaviors, and weight as a result of program use. | *Experienced changes*  *Impact on eating* |  | “Before I started using the app, I wouldn’t really think about it. I’d pause whatever I was doing, and I would go into the kitchen and just look for food without any real thought process, I want to eat. The app forced me to…Say I was watching a show, I like to binge-watch shows. I would pause the show and I’d be like EAT, well wait why? Well, why eat? And then be like okay, bored more so than hungry.” (5346, 34 y/o, lesbian, genderqueer)  "Simply just to slow down and really think about why I’m eating, if I’m really hungry or thirsty, if I just need to ride out the cravings. Having a bite or two of ice cream instead of a bowl is fine. Or if I’m craving sweets, I can have an apple. Those tips that came through on the app were really helpful." (2411, 41 y/o, bisexual, female)  “I think the other piece that was really big was like getting in touch with the feelings of discomfort that come with overeating like that disenchantment part which is so like logical like duh. I don't think I had really spent time feeling what that's physically like in my body versus just like guilt or shame or judgment.” (8587, 38 y/o, bisexual, female) |
| Improvements in mental health and well-being by completing the program. | *Impact on well-being*  *Impact on mental health* |  | “Yeah, I think the app gave me my own space to work on myself and my health. That’s something I really needed – a sacred space for myself and my mental health – my own space to continue on the things that I need to do for myself.” (2794, 52 y/o, pansexual, androgenous)  "It definitely helped me kind of boost my confidence, a little bit and but I’m not sure if that’s related to you know me being queer or just overall." (9648, 49 y/o, bisexual, female)  “It helped me with my thoughts and my anxiety. It like helps and the breathing techniques and stuff like that helps me and it helps me focus on myself and what was going on with myself.” (2517, 41 y/o, bisexual, female) |
